# Pharyngeal Airway Changes After Functional Orthodontic Treatment in Growing Class II Patients: A Retrospective Cephalometric Comparison of Twin Block, RPE and AMCOP^®^

**DOI:** 10.3390/life15121939

**Published:** 2025-12-18

**Authors:** Alessio Danilo Inchingolo, Angelo Michele Inchingolo, Irene Palumbo, Daniela Di Venere, Cinzia Maspero, Francesco Inchingolo, Filippo Cardarelli, Grazia Marinelli, Gianna Dipalma

**Affiliations:** 1 Department of Interdisciplinary Medicine, University of Bari “Aldo Moro”, 70121 Bari, Italy; alessiodanilo.inchingolo@uniba.it (A.D.I.); irenepalu@icloud.com (I.P.); daniela.divenere@uniba.it (D.D.V.); francesco.inchingolo@uniba.it (F.I.); drfilippocardarelli@libero.it (F.C.); grazia.marinelli@live.it (G.M.); 2 Department of Biomedical, Surgical and Dental Sciences, University of Milan, 20100 Milan, Italy; cinzia.maspero@unimi.it; 3 Fondazione IRCCS Cà Granda, Ospedale Maggiore Policlinico, 20100 Milan, Italy

**Keywords:** class II malocclusion, mandibular advancement, functional orthodontic appliances, pharyngeal airway, twin block, rapid palatal expander, AMCOP^®^ device, cephalometric analysis

## Abstract

Pharyngeal airway morphology is closely linked to craniofacial development, and children with Class II malocclusion—often characterized by mandibular retrusion—may present reduced airway dimensions and a higher risk of obstructive sleep apnea. This retrospective single-center study evaluated whether functional orthodontic appliances can improve pharyngeal airway space by promoting mandibular advancement during growth. Fifty patients aged 6–12 years with skeletal Class II malocclusion (ANB > 4°) were treated with a Twin Block appliance (*n* = 18), Rapid Palatal Expander (RPE; *n* = 16), or AMCOP^®^ elastodontic device (*n* = 16). Pre- and post-treatment lateral cephalograms were analyzed to assess skeletal (SNA, SNB, ANB, Co–Gn), dentoalveolar (overjet, overbite, IMPA), and pharyngeal airway variables (SPAS, MAS, PAS). Intra-group changes were tested with paired t-tests and inter-group differences with one-way ANOVA and Tukey post hoc tests (α = 0.05). All appliances produced statistically significant increases in pharyngeal airway dimensions. The Twin Block group showed the greatest improvements, with mean increases of 2.1 mm in SPAS (*p* < 0.001), 1.8 mm in MAS (*p* < 0.001), and 1.5 mm in PAS (*p* < 0.001), together with a significant mandibular advancement (ΔSNB = +1.7°; ΔANB = −1.5°) and elongation of mandibular length (ΔCo–Gn = +3.3 mm). RPE and AMCOP^®^ induced more moderate, yet significant, skeletal and airway changes (RPE: SPAS +1.4 mm, *p* = 0.006; MAS +0.9 mm, *p* = 0.009; PAS +0.8 mm, *p* = 0.022; AMCOP^®^: SPAS +0.9 mm, *p* = 0.034; MAS +0.9 mm, *p* = 0.041; PAS +0.6 mm, *p* = 0.037). Within the limitations of this small, retrospective single-center sample, the findings indicate that functional orthodontic treatment during growth may be associated with increases in pharyngeal airway dimensions in Class II patients. Among the appliances evaluated, the Twin Block showed the most pronounced skeletal and morphological airway changes.

## 1. Introduction

The morphology of the pharyngeal airway represents a crucial determinant of craniofacial growth, respiratory efficiency, and overall health in pediatric patients [1,2,3,4,5,6]. Alterations in airway dimensions are often associated with skeletal discrepancies of the craniofacial complex, particularly those involving mandibular retrusion [1,7,8,9,10,11,12,13]. Among these, Class II malocclusion is one of the most common dentoskeletal disharmonies observed in children and adolescents and is frequently characterized by mandibular deficiency, increased overjet, and a convex facial profile [6,13,14,15,16,17,18,19,20,21].

Class II prevalence varies considerably across age groups, populations, and diagnostic criteria. Global estimates report an average prevalence of approximately 24% in children and adolescents, with values ranging from 1% to over 40% [22,23,24]. In the United States, about 15% of the general population presents with Class II features, while studies in Asian populations—such as Chinese preschool and school-age children—show rates between 8% and 10% [25,26]. In Italy, epidemiological data indicate a prevalence ranging from 24% to nearly 47% in the pediatric population, depending on region and study design [27,28]. These variations reflect the multifactorial etiology of Class II malocclusion and its early onset, often emerging in the primary dentition and potentially persisting or worsening during growth.

These anatomical features can contribute to a reduced pharyngeal airway space, predisposing affected individuals to functional impairments, such as mouth breathing and obstructive sleep apnea (OSA) [7,29,30,31,32,33,34,35].

OSA is a sleep disorder characterized by recurrent partial or complete collapse of the upper airway during sleep, leading to episodes of apnea or hypopnea lasting ≥10 s and accompanied by oxygen desaturation and sleep fragmentation [36,37,38,39,40,41,42]. Diagnosis relies on the apnea–hypopnea index (AHI), with adult severity classified as mild (5–14 events/h), moderate (15–29 events/h), and severe (≥30 events/h) [43,44,45,46,47]. OSA is commonly associated with loud snoring, witnessed apneas, non-restorative sleep, and excessive daytime sleepiness, and is strongly linked to obesity, male sex, older age, and craniofacial or upper-airway abnormalities [48,49,50,51,52,53,54]. The disorder increases the risk of hypertension, type 2 diabetes, cardiovascular disease, stroke, and all-cause mortality. In children, the clinical presentation differs and includes habitual snoring, restless sleep, nocturnal enuresis, morning headaches, and prominent neurocognitive or behavioral disturbances rather than daytime sleepiness. Pediatric OSA is diagnosed using lower AHI thresholds (abnormal ≥1 event/h), reflecting increased vulnerability to neurocognitive and cardiometabolic sequelae [3,4,7,55].

In recent decades, numerous studies have highlighted the close relationship between craniofacial morphology and upper airway patency [2,4,28,49,55,56,57,58]. A reduced anteroposterior dimension of the mandible and an increased ANB angle have been associated with narrower pharyngeal spaces, particularly at the oropharyngeal and hypopharyngeal levels [2,59]. Such features can impair airflow during sleep and are increasingly recognized as contributing risk factors for pediatric OSA [60,61,62,63,64,65,66]. The condition, characterized by recurrent episodes of partial or complete airway collapse, has been linked to neurocognitive, cardiovascular, and behavioral complications in children [32,67,68,69,70,71,72,73].

Given these associations, orthodontic interventions that promote mandibular growth and improve jaw positioning have been proposed not only for dentoskeletal correction but also as adjunctive therapies to enhance airway function [29,39,49,67,74,75,76,77]. Functional orthopedic appliances act through mandibular advancement and muscular adaptation, thereby influencing the position of the tongue, soft palate, and pharyngeal walls [5,17,78]. These adaptive changes can lead to measurable increases in airway volume and cross-sectional area, particularly when treatment is initiated during the growth phase, when skeletal plasticity is maximal [42,54,63,79,80,81,82,83]. Prospective clinical evidence has demonstrated that orthodontic interventions can alter airway dimensions in patients with OSA, underscoring the clinical relevance of skeletal and functional changes during treatment [84].

**Figure 1:**
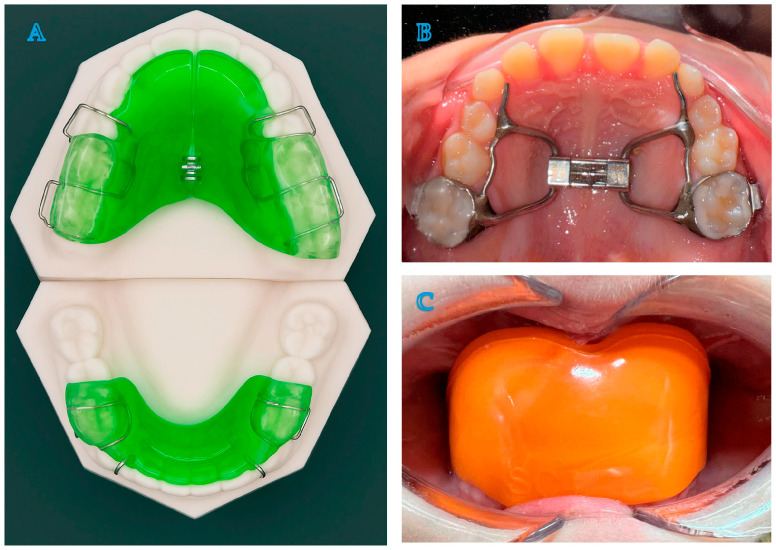
Twin Block (**A**), Rapid Palatal Expander (**B**) and AMCOP^®^ SC (**C**).

Among functional devices, the Twin Block (TB) appliance is widely used for mandibular advancement in growing patients with Class II malocclusion [85,86,87,88,89,90,91]. Its design allows continuous forward posturing of the mandible, resulting in both skeletal remodeling and dentoalveolar adaptation [71,91,92,93,94,95,96]. The Rapid Palatal Expander (RPE), primarily used for transverse maxillary deficiency, may also indirectly increase nasal and pharyngeal airway dimensions by expanding the midpalatal suture and improving nasal airflow [73,97,98,99,100,101,102,103,104,105] The rapid palatal expander (RPE) was included as a comparison group because it represents one of the most commonly used interceptive appliances in growing patients. Although RPE does not advance the mandible, maxillary transverse expansion may indirectly influence airway configuration by increasing nasal cavity volume, reducing nasal resistance, and promoting more favorable tongue posture. Given its widespread clinical use and its potential indirect effects on upper airway morphology, RPE provides a relevant comparative framework for evaluating different treatment approaches in growing Class II patients. More recently, elastodontic appliances such as AMCOP^®^ have been introduced as interceptive devices to support craniofacial growth through soft, medical-grade silicone components designed to promote neuromuscular balance, improved tongue posture, and functional adaptation with relatively low compliance requirements. The AMCOP^®^ system (Protection Smile, Capurso (BA), Italy) applies light elastic forces to guide dentoalveolar development and harmonize orofacial muscle activity during growth [63,106,107,108,109,110] (Figure 1).

Despite growing clinical use of these appliances, comparative data regarding their effects on pharyngeal airway morphology remain limited. Previous research has often focused on individual devices or restricted parameters, without a comprehensive assessment of skeletal, dentoalveolar, and pharyngeal outcomes within the same cohort. Furthermore, the extent to which mandibular advancement correlates with changes in specific airway regions—such as the superior posterior airway space (SPAS), middle airway space (MAS), and posterior airway space (PAS)—has not been fully elucidated.

**Figure 2:**
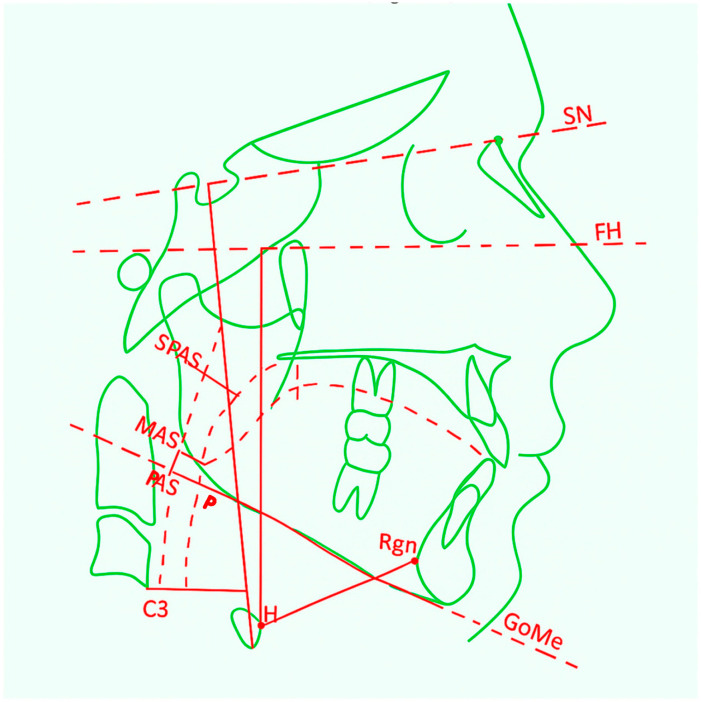
Lateral cephalometric tracing highlighting craniofacial reference planes and the pharyngeal airway measurements SPAS, MAS, and PAS used to assess upper-airway morphology.

SPAS represents the linear distance between the posterior margin of the soft palate and the posterior pharyngeal wall, reflecting the caliber of the retropalatal region [111,112]. MAS corresponds to the oropharyngeal width at the level of the tongue body, while PAS measures the distance between the base of the tongue and the posterior pharyngeal wall, representing the retroglossal compartment [113,114,115,116]. Reductions in these measurements are commonly associated with mandibular retrusion and an increased susceptibility to upper airway collapse during sleep [9,30,59,111,115,117,118]. The analysis of SPAS, MAS, and PAS therefore provides an objective and reproducible method to evaluate morphological changes in the pharyngeal airway and to assess the impact of functional orthopedic treatments aimed at mandibular advancement (Figure 2).

The present study aimed to evaluate the pharyngeal airway changes following mandibular advancement induced by three different orthodontic appliances—TB, RPE, and AMCOP^®^ elastodontic device—using cephalometric analysis on lateral teleradiographs. Secondary objectives included assessing the relationship between skeletal modifications and airway dimensional changes, and determining which treatment modality produced the most significant improvement in pharyngeal airway patency.

## 2. Materials and Methods

This study was designed as a retrospective, observational, single-center analysis conducted at the Orthodontics Department of the University Hospital “Aldo Moro” of Bari (Italy). The research aimed to assess the cephalometric variations in the pharyngeal airway following mandibular advancement induced by different orthodontic appliances in pediatric patients. The study was conducted in accordance with the Declaration of Helsinki and approved by the Local Ethics Committee IRCCS Istituto Oncologico “Gabriella Serio” and obtained favorable approval (Prot. n. 969 del 1 October 2025).

### 2.1. Sample Selection

The study population consisted of 50 pediatric patients (27 females, 23 males) aged 6 to 12 years (mean age 9.2 ± 1.8 years), who underwent orthodontic treatment with one of the following appliances:

- Group A: Twin Block appliance (custom-made removable functional appliance fabricated in the orthodontic laboratory of the Orthodontics Department, University Hospital “Aldo Moro”, Bari, Italy; *n* = 18).
- Group B: Rapid Palatal Expander (custom-made fixed appliance fabricated in the same institutional orthodontic laboratory; *n* = 16).
- Group C: AMCOP^®^ elastodontic device (Protection Smile S.r.l., Capurso (BA), Italy; *n* = 16).

A total of 50 consecutive patients were retrospectively selected from the institutional archive based on the predefined inclusion and exclusion criteria. No matching procedures were applied; however, baseline comparability among the three treatment groups (TB, RPE, AMCOP^®^) was assessed. The groups did not differ significantly in age, sex distribution, skeletal severity (ANB, SNB), or treatment duration at T0 (*p* > 0.05 for all comparisons). Each patient had complete diagnostic documentation, including lateral cephalometric radiographs acquired before treatment (T0) and after treatment (T1). All cephalograms were taken by the same operator using standardized positioning and exposure parameters. All lateral cephalograms were obtained with patients in natural head position, teeth in maximum intercuspation, and lips relaxed. Patients were instructed to breathe gently through the nose during acquisition to minimize airway variability related to respiratory phase. Tongue position was not actively manipulated but patients were asked to maintain a relaxed posture. Radiographic and clinical records of eligible patients were retrospectively retrieved from the department archives. All data were pre-existing and had been acquired during routine orthodontic care. Cephalometric tracing and data analysis were performed after ethical approval, in October 2025.

#### 2.1.1. Inclusion Criteria

- Age between 6 and 12 years at T0.
- Availability of high-quality pre- and post-treatment lateral cephalometric radiographs.
- Class II skeletal malocclusion defined by ANB > 4°.
- Good general health, with no systemic or respiratory conditions known to influence craniofacial growth or upper airway morphology.
- Treatment performed with TB, RPE, or AMCOP^®^ appliances.

#### 2.1.2. Exclusion Criteria

- Craniofacial syndromes, congenital malformations, or genetic disorders;
- Incomplete or poor-quality radiographic documentation;
- Ongoing respiratory or neuromuscular conditions influencing airway dimensions;
- Previous orthognathic surgery or concurrent orthodontic treatment with fixed appliances;
- Lack of consent for data use.

#### 2.1.3. Twin Block (Group A)

The TB appliance consisted of upper and lower acrylic plates with occlusal inclined planes designed to posture the mandible forward. The construction bite was taken with an individualized mandibular advancement ranging from 5 to 7 mm, depending on the skeletal discrepancy, and a 2–4 mm increase in vertical dimension.

Patients were instructed to wear the appliance full time (20–22 h/day), removing it only for meals and oral hygiene.

Compliance was monitored at each follow-up visit (every 4–6 weeks) through parental reporting and clinical evaluation of wear marks and appliance seating.

Active treatment continued until sagittal correction and overjet normalization were achieved, generally over a period of 10–12 months.

#### 2.1.4. Rapid Palatal Expander (Group B)

In the RPE group, banded Hyrax-type expander was anchored to the upper premolars and molars. After cementation, the screw was activated at a rate of one quarter-turn per day until achievement of the planned expansion and the appearance of a midline diastema. The appliance was kept in place as a passive retainer for at least 6 months to allow stabilization of the skeletal expansion.

#### 2.1.5. AMCOP^®^ Elastodontic Device (Group C)

Patients in Group C were treated with AMCOP^®^ SC elastodontic devices made of medical-grade silicone and designed to promote neuromuscular balance, dentoalveolar alignment, and mild mandibular advancement.

Patients were instructed to wear the device overnight (8–10 h) and for an additional 1–2 h during the day.

Compliance was monitored through parental reports and clinical checks at follow-up visits scheduled every 4–6 weeks.

The average treatment duration was approximately 10–12 months.

Treatment duration was recorded for each patient as the interval between appliance delivery (T0) and completion of active therapy (T1). Mean treatment duration was 11.0 ± 0.8 months for the Twin Block group, 9.0 ± 0.8 months for the RPE group, and 11.0 ± 0.8 months for the AMCOP^®^ group.

### 2.2. Cephalometric Analysis

Cephalometric analysis was performed on digital lateral teleradiographs (LL) using Delta Dent software version 2.2.1, (Outside Format, Via Circonvallazione D, 28, 26025 Pandino (CR), Italy), a CE-certified medical device compliant with EU Regulation 2017/745 (MDR) and GDPR (EU 2016/679). The software allows high-precision manual landmark identification and angular and linear measurements of craniofacial structures.

Each cephalogram was traced by a single trained examiner to ensure consistency. The examiner performing the tracings was not blinded to treatment group or timepoint, which may introduce measurement bias. To assess intra-examiner reliability, 20 cephalograms were retraced after two weeks. ICC values ranged from 0.86–0.94 for skeletal variables and 0.83–0.91 for airway variables, indicating good to excellent reliability. The following skeletal, dentoalveolar, and pharyngeal airway parameters were measured:

#### 2.2.1. Skeletal Parameters

- SNA (°): Anteroposterior position of the maxilla relative to the cranial base (mean = 82° ± 2).
- SNB (°): Anteroposterior position of the mandible relative to the cranial base (mean = 80° ± 2).
- ANB (°): Skeletal discrepancy between maxilla and mandible (mean = 2° ± 2).
- Co–Gn (mm): Total mandibular length from condylion to gnathion.
- FMA (°): Facial divergence according to Tweed (mean = 25° ± 3).
- SN–GoGn (°): Total divergence (mean = 32° ± 5).
- ArGo–GoMe (°): Gonial angle, indicating mandibular morphology.

#### 2.2.2. Dentoalveolar Parameters

- Overjet (mm): Horizontal overlap between upper and lower incisors (mean = 2.5 ± 2.5 mm).
- Overbite (mm): Vertical overlap between upper and lower incisors (mean = 2.5 ± 2.5 mm).
- IMPA (°): Inclination of lower incisors relative to the mandibular plane (mean = 90° ± 5).

#### 2.2.3. Pharyngeal Airway Parameters (Table 1)

- SPAS (Superior Posterior Airway Space): Distance between the posterior margin of the soft palate and the posterior pharyngeal wall.
- MAS (Middle Airway Space): Distance at the level of the oropharynx.
- PAS (Posterior Airway Space): Distance between the base of the tongue and the posterior pharyngeal wall.

**Table 1:** Definitions of cephalometric landmarks and airway measurements.

Measurement	Definition	Measurement Technique
SPAS (Superior Posterior Airway Space)	Linear distance between the posterior border of the soft palate and the posterior pharyngeal wall.	Measured along a line perpendicular to the soft palate at the level of the widest portion of the velopharyngeal airway.
MAS (Middle Airway Space)	Linear distance between the posterior border of the tongue (at the level of the base of the tongue) and the posterior pharyngeal wall.	Measured on the line passing through the midpoint of the soft palate and oriented parallel to the mandibular plane, intersecting the oropharyngeal airway.
PAS (Inferior Posterior Airway Space)	Linear distance between the base of the tongue (near the epiglottis) and the posterior pharyngeal wall.	Measured along a line parallel to the mandibular plane at the level of the intersection between the tongue base and the posterior airway space.

All measurements were obtained at both T0 and T1 to evaluate skeletal, dental, and pharyngeal variations after treatment.

### 2.3. Statistical Analysis

Data were organized in a dedicated spreadsheet and analyzed using SPSS software (version 27.0, IBM Corp., Armonk, NY, USA). Descriptive statistics (mean ± standard deviation) were calculated for all variables, and intra-group changes between T0 and T1 were assessed using paired t-tests. Inter-group comparisons among the TB, RPE, and AMCOP^®^ treatment groups were performed through one-way ANOVA followed by Tukey’s post-hoc test. A *p*-value < 0.05 was considered statistically significant. Effect sizes (Cohen’s d) were calculated for all intra-group pre–post changes in pharyngeal airway dimensions (SPAS, MAS, PAS) by dividing the mean T1–T0 difference by the pooled standard deviation of the pre- and post-treatment measurements.

## 3. Results

The study sample consisted of 50 pediatric patients (27 females, 23 males; mean age 9.2 ± 1.8 years) divided into three treatment groups: TB (*n* = 18), RPE (*n* = 16), and AMCOP^®^ (*n* = 16). All patients completed the full treatment phase with comparable pre-treatment characteristics (no statistically significant differences at T0 among groups, *p* > 0.05).

### 3.1. Skeletal Changes

Following treatment, all groups demonstrated significant improvements in mandibular position and sagittal relationships, with the TB group exhibiting the most pronounced skeletal changes (Table 2).

The TB treatment significantly advanced the mandible (ΔSNB = +1.7°, *p* < 0.001; ΔANB = −1.5°, *p* < 0.001), with a concurrent increase in total mandibular length (ΔCo–Gn = +3.3 mm). RPE and AMCOP^®^ also induced modest but statistically significant skeletal improvements, albeit less pronounced (*p* < 0.05).

### 3.2. Dentoalveolar Changes (Table 3)

All appliances produced a reduction in overjet and overbite, with the TB showing the greatest improvement (−3.6 mm in overjet, *p* < 0.001). A mild proclination of lower incisors (IMPA +2.9°) was also observed.

**Table 3:** Dentoalveolar parameters (mean ± SD).

Parameter	Group	T0	T1	Δ (T1–T0)	*p*-Value
Overjet (mm)	TB (*n* = 18)	6.7 ± 1.4	3.1 ± 1.2	−3.6	<0.001
	RPE (*n* = 16)	6.5 ± 1.5	4.9 ± 1.3	−1.6	0.012
	AMCOP^®^ (*n* = 16)	6.4 ± 1.6	4.7 ± 1.2	−1.7	0.009
Overbite (mm)	TB (*n* = 18)	3.8 ± 1.1	2.4 ± 1.0	−1.4	0.014
	RPE (*n* = 16)	3.6 ± 1.0	3.0 ± 1.0	−0.6	0.093
	AMCOP^®^ (*n* = 16)	3.7 ± 1.2	2.9 ± 1.1	−0.8	0.081
IMPA (°)	TB (*n* = 18)	91.2 ± 4.0	94.1 ± 3.8	+2.9	0.019
	RPE (*n* = 16)	90.8 ± 4.1	92.3 ± 3.9	+1.5	0.046
	AMCOP^®^ (*n* = 16)	91.0 ± 3.8	92.5 ± 3.7	+1.5	0.052

### 3.3. Pharyngeal Airway Changes

All treatment modalities resulted in statistically significant enlargement of pharyngeal airway spaces, with the TB appliance showing the most substantial mean increases across all parameters (SPAS +2.1 mm, MAS +1.8 mm, PAS +1.5 mm; *p* < 0.001). RPE and AMCOP^®^ also produced significant but less marked improvements. In terms of effect size, the Twin Block group showed large changes in all airway variables (SPAS d = 1.35; MAS d = 1.24; PAS d = 1.11). The RPE group exhibited moderate-to-large effects (SPAS d = 0.84; MAS d = 0.76; PAS d = 0.59), whereas the AMCOP^®^ group showed small-to-moderate effects (SPAS d = 0.55; MAS d = 0.48; PAS d = 0.46).

Pre–post changes in pharyngeal airway dimensions, together with 95% confidence intervals and effect sizes, are reported in Table 4.

In the Twin Block group, SPAS, MAS and PAS increased by +2.14 mm [1.92; 2.36], +1.96 mm [1.81; 2.12], and +1.42 mm [1.27; 1.57], respectively, all supported by large effect sizes (d = 1.11–1.35).

RPE showed moderate but significant increases, with SPAS +1.41 mm [1.15; 1.67], MAS +0.89 mm [0.67; 1.10], and PAS +0.75 mm [0.47; 1.04], consistent with effect sizes in the moderate range (d = 0.59–0.84).

The AMCOP^®^ group demonstrated smaller but still significant dimensional increases, with confidence intervals consistently above zero (SPAS +0.85 mm [0.60; 1.10], MAS +0.91 mm [0.73; 1.09], PAS +0.58 mm [0.36; 0.81]).

Treatment duration differed slightly among groups, with the Twin Block and AMCOP^®^ therapies lasting on average 11.0 ± 0.8 months, while RPE treatment had a shorter duration of 9.0 ± 0.8 months.

### 3.4. Intergroup Comparison

One-way ANOVA revealed significant intergroup differences for SNB, ANB, Co–Gn, and all pharyngeal airway parameters (*p* < 0.05).

Post hoc analysis (Tukey test) confirmed that TB achieved significantly greater increases in SNB and airway dimensions (SPAS, MAS, PAS) compared with both RPE and AMCOP^®^ (*p* < 0.01). Differences between RPE and AMCOP^®^ were not statistically significant (*p* > 0.05).

## 4. Discussion

The present study investigated the pharyngeal airway changes following mandibular advancement obtained with three orthodontic appliances—TB, RPE, and AMCOP^®^ elastodontic device—in a sample of growing patients with skeletal Class II malocclusion. The cephalometric analysis demonstrated significant increases in the upper airway dimensions across all treatment groups, with the most pronounced changes observed in patients treated with the TB appliance.

These findings are consistent with previous literature highlighting the close association between mandibular position and pharyngeal airway patency [50,117,118,119,120,121,122,123,124,125,126,127]. Several authors have reported that mandibular retrusion contributes to narrowing of the retroglossal and retropalatal spaces, predisposing to narrowing of the retroglossal and retropalatal spaces, which has been associated in the literature with compromised airway dimensions in growing patients [60,68,128,129,130,131]. Functional orthopedic therapy, by advancing the mandible and repositioning the tongue anteriorly, can therefore be associated with enlargement of the pharyngeal airway; however, functional effects cannot be inferred from morphological data alone [4,132,133,134,135,136,137].

### 4.1. Skeletal Modifications and Airway Response

In the current study, the TB appliance produced a mean increase in SNB of +1.7°, accompanied by a reduction in ANB of −1.5°, confirming its effectiveness in sagittal correction of Class II malocclusion through true mandibular advancement [70,79,122,123,138,139,140,141,142,143,144,145,146]. The observed elongation of the mandibular body (ΔCo–Gn = +3.3 mm) further supports the skeletal contribution of functional treatment during the growth period.

These skeletal changes were paralleled by marked airway expansion, particularly at the oropharyngeal level. The mean increases in SPAS (+2.1 mm), MAS (+1.8 mm), and PAS (+1.5 mm) observed in the TB group suggest that the anterior repositioning of the mandible may be accompanied by forward displacement tendencies of the tongue and soft tissues, as described in the previous literature. These results align with those reported by Ozbek et al. (1998), Jena et al. (2006) and other authors, who documented significant enlargement of the superior and middle airway spaces following TB therapy in growing subjects [79,112,147,148,149,150,151,152,153,154]. Although the airway increases observed in the Twin Block group (e.g., SPAS +2.1 mm) were statistically significant and supported by large effect sizes, their clinical relevance remains uncertain. Linear cephalometric measurements reflect only projected morphological changes and do not allow conclusions regarding airflow, airway resistance, or sleep-disordered breathing. Therefore, the dimensional increases reported in this study should be interpreted as morphological rather than functional modifications.

The RPE group exhibited moderate but significant increases in airway dimensions (SPAS +1.3 mm; MAS +1.1 mm; PAS +0.8 mm). The expansion of the maxillary arch achieved by RPE widens the maxillary arch and nasal cavity, and previous studies have proposed secondary effects on nasal airflow and tongue posture, although such mechanisms were not evaluated in the present study. These findings corroborate those of Pirelli et al. (2021) and Fastuca et al. (2015) and other authors, who demonstrated improvements in nasal resistance and airway volume following rapid palatal expansion in children with constricted maxillae [155,156,157,158,159,160]. It is essential to consider the different biomechanical actions of the appliances evaluated. The Twin Block and AMCOP^®^ devices primarily influence sagittal mandibular relationships and may be associated with morphological changes along the anteroposterior dimension of the airway. In contrast, the rapid palatal expander (RPE) acts mainly in the transverse plane. Its potential contribution to airway morphology results from maxillary expansion and associated changes in nasal airflow rather than from sagittal correction. Consequently, inter-group comparisons should be interpreted with caution, as the underlying mechanisms differ substantially and are expected to produce morphological changes in different magnitude and regional distribution. The inclusion of RPE in this study nonetheless reflects its widespread clinical use in interceptive orthodontics and its relevance as a complementary approach in the early management of growing patients with skeletal discrepancies or airway-related dysfunctions.

The AMCOP^®^ group showed smaller yet statistically significant improvements in airway spaces (SPAS +0.9 mm; MAS +0.7 mm; PAS +0.6 mm). Elastodontic appliances such as AMCOP^®^ function primarily through neuromuscular re-education and light biomechanical forces, promoting balanced growth and functional remodeling rather than substantial skeletal advancement as already described [63,106,107,108,109].

Although our results indicate statistically significant increases in SPAS, MAS, and PAS, these findings should be interpreted with caution due to the intrinsic limitations of two-dimensional lateral cephalograms. Linear measurements obtained on 2D images represent only a sagittal projection of a three-dimensional structure and cannot account for airway volume, cross-sectional morphology, or asymmetries. Functional appliances may influence soft-tissue positioning and airway morphology, although such qualitative changes cannot be assessed using 2D cephalograms. In this context, cone-beam computed tomography (CBCT) would allow a more comprehensive evaluation, including three-dimensional volumetry, minimum cross-sectional areas, and localized constrictions, and would therefore provide a more accurate representation of real airway changes. From a radiological and ethical perspective, the choice of 2D imaging in the present retrospective cohort reflects the fact that lateral cephalograms are part of routine orthodontic records and are associated with lower radiation exposure in growing patients. Cone-beam computed tomography (CBCT) provides superior three-dimensional information on airway volume and minimum cross-sectional areas and would certainly be desirable in future prospective investigations. However, in the context of the current study, systematic CBCT acquisition would have required a second-level imaging procedure performed exclusively for research purposes, which was not justified in asymptomatic pediatric patients. Accordingly, the present 2D analysis should be regarded as a first-level morphological evaluation and as a hypothesis-generating step toward more detailed CBCT-based studies.

### 4.2. Comparison Among Treatment Modalities

The intergroup comparison confirmed that the TB appliance produced significantly greater skeletal and linear airway modifications than both RPE and AMCOP^®^ (*p* < 0.01). These findings suggest that the magnitude of mandibular advancement is associated with the extent of morphological airway changes. In contrast, appliances without true sagittal correction, such as RPE or elastodontic therapy, tend to produce smaller linear changes, reflecting their different biomechanical modes of action. From a clinical standpoint, the present findings support the idea that early identification and management of mandibular retrusion may represent an important aspect of the overall evaluation of children presenting with airway-related dysfunctions such as mouth breathing or habitual snoring. Although functional orthopedic treatment primarily aims to correct skeletal discrepancies, the associated morphological changes in upper airway dimensions could contribute to more favorable airway conditions. Recent CBCT studies have highlighted relationships between sagittal skeletal discrepancies and adjacent airway related structures, supporting the value of three dimensional assessment when feasible [161]. However, because the current study assessed only structural parameters and did not include functional respiratory or sleep-related measurements, any potential impact on breathing efficiency or long-term risks such as pediatric OSA cannot be confirmed and should be explored in future prospective studies integrating objective functional outcomes [162,163,164,165,166,167].

### 4.3. Interpretation and Clinical Implications

Previous research has shown that mandibular advancement can modify the spatial configuration of the upper airway by influencing the position of the tongue and hyoid bone. The present findings are consistent with this concept and provide quantitative evidence of morphological enlargement of pharyngeal airway spaces across different appliances. However, these changes must not be interpreted as direct functional improvements, as no respiratory or sleep-related assessments were performed [4,49,56,70,168,169]. Within this morphological framework, the TB appliance demonstrated the greatest skeletal and airway changes, aligning with its role in sagittal correction. RPE and AMCOP^®^ may serve as complementary or interceptive modalities when skeletal discrepancies coexist with transverse maxillary constriction or neuromuscular imbalance, but their airway effects remain predominantly structural and device-specific. Further prospective studies incorporating three-dimensional imaging and functional respiratory evaluations are required to determine whether these morphological changes translate into clinically meaningful improvements.

### 4.4. Limitations

This study presents several limitations. First, the retrospective single-center design inevitably introduces potential selection bias and prevents strict control over treatment initiation, duration, follow-up intervals, and compliance monitoring. Patient adherence to appliance wear—particularly for the Twin Block and AMCOP^®^ devices—could not be objectively verified and may have influenced the magnitude of the observed skeletal and airway changes. Moreover, the absence of an untreated control group limits the ability to distinguish treatment-related effects from normal growth, although ethical considerations prevent prolonged withholding of treatment in growing patients. Second, the exclusive use of two-dimensional lateral cephalometry, while common in orthodontic diagnostics, restricts the evaluation of airway morphology to linear sagittal measurements and does not permit assessment of volumetric, cross-sectional, or asymmetrical characteristics. Three-dimensional imaging modalities such as CBCT would provide a more comprehensive evaluation but were not justified in this pediatric retrospective cohort due to ethical concerns regarding radiation exposure. Third, the morphometric nature of the data prevents any direct conclusions regarding functional respiratory outcomes. Improvements in SPAS, MAS, and PAS do not necessarily correspond to better airflow dynamics or reduced sleep-disordered breathing, and no respiratory or polysomnographic parameters (e.g., apnea–hypopnea index, oxygen saturation) were available for correlation. Additionally, all cephalometric tracings were performed by a single, non-blinded examiner, which may introduce measurement bias despite high intra-examiner reliability. The lack of long-term follow-up further limits the ability to determine the stability of the observed airway modifications.

Future research should include prospective, longitudinal studies incorporating 3D imaging modalities, objective compliance monitoring, and functional respiratory assessments to better clarify the clinical relevance and persistence of the morphological changes reported in the present study.

## 5. Conclusions

Functional orthodontic therapy aimed at mandibular advancement has been associated with enlargement of pharyngeal airway dimensions in growing patients with Class II malocclusion. Among the different appliances, the Twin Block demonstrated the most marked skeletal and airway changes, reflecting its recognized effectiveness in stimulating mandibular growth. The RPE produced a more moderate increase in airway dimensions, which is consistent with its transverse mode of action and its potential indirect influence on maxillary and upper airway morphology. The AMCOP^®^ elastodontic device, although inducing smaller modifications, still achieved measurable changes compatible with its functional and neuromuscular characteristics. Overall, early orthodontic intervention targeting mandibular retrusion may contribute to creating more favorable upper airway conditions in growing patients, although the extent of this influence can vary depending on the appliance and its biomechanical action. Within the limitations of this small retrospective sample and the use of 2D cephalometric measurements, the present findings indicate an association between functional orthopedic treatment and linear morphological increases in pharyngeal airway dimensions. These results should be interpreted as preliminary morphological observations, and future prospective studies with larger samples and functional respiratory assessments are required to confirm their clinical relevance. In addition to skeletal mandibular advancement, several dentoalveolar adaptations may have contributed to the airway changes observed in this study. Functional appliances are known to induce varying degrees of lower incisor proclination and modifications in vertical facial dimension, which can alter the position of the tongue and the available oral cavity space. Such changes may indirectly influence the morphology of the upper airway by facilitating a more anterior resting position of the tongue and reducing posterior crowding of soft tissues. Furthermore, improvements in neuromuscular balance and postural tone—particularly relevant in elastodontic therapy—may also promote functional adaptations of the oropharyngeal region. These dentoalveolar and functional mechanisms should therefore be considered alongside skeletal effects when interpreting the airway modifications associated with the different treatment modalities.

## Figures and Tables

**Table 2:** Skeletal cephalometric parameters (mean ± SD).

Parameter	Group	T0	T1	Δ (T1–T0)	*p*-Value
SNA (°)	TB (*n* = 18)	81.6 ± 2.1	81.9 ± 2.0	+0.3	0.147
	RPE (*n* = 16)	82.0 ± 1.9	82.2 ± 1.8	+0.2	0.210
	AMCOP^®^ (*n* = 16)	81.8 ± 2.0	81.9 ± 1.9	+0.1	0.324
SNB (°)	TB (*n* = 18)	77.8 ± 1.9	79.5 ± 1.8	+1.7	<0.001
	RPE (*n* = 16)	78.1 ± 1.8	78.8 ± 1.7	+0.7	0.032
	AMCOP^®^ (*n* = 16)	77.9 ± 1.7	78.5 ± 1.6	+0.6	0.041
ANB (°)	TB (*n* = 18)	3.8 ± 1.2	2.3 ± 1.1	−1.5	<0.001
	RPE (*n* = 16)	3.9 ± 1.1	3.2 ± 1.0	−0.7	0.028
	AMCOP^®^ (*n* = 16)	4.0 ± 1.3	3.4 ± 1.2	−0.6	0.037
Co–Gn (mm)	TB (*n* = 18)	97.5 ± 3.6	100.8 ± 3.8	+3.3	<0.001
	RPE (*n* = 16)	97.8 ± 3.7	99.0 ± 3.6	+1.2	0.045
	AMCOP^®^ (*n* = 16)	98.1 ± 3.4	99.4 ± 3.5	+1.3	0.036

**Table 4:** Pharyngeal airway parameters (mean ± SD).

Parameter	Group	T0 Mean ± SD	T1 Mean ± SD	Mean Δ (T1–T0)	95% CI for Δ	*p*-Value	Cohen’s d
SPAS (mm)	TB (*n* = 18)	9.8 ± 1.5	11.9 ± 1.6	+2.14	[1.92; 2.36]	<0.001	1.35
	RPE (*n* = 16)	9.9 ± 1.6	11.2 ± 1.5	+1.41	[1.15; 1.67]	0.006	0.84
	AMCOP^®^ (*n* = 16)	9.7 ± 1.7	10.6 ± 1.6	+0.85	[0.60; 1.10]	0.034	0.55
MAS (mm)	TB	10.2 ± 1.4	12.0 ± 1.5	+1.96	[1.81; 2.12]	<0.001	1.24
	RPE	10.3 ± 1.5	11.4 ± 1.4	+0.89	[0.67; 1.10]	0.009	0.76
	AMCOP^®^	10.1 ± 1.5	10.8 ± 1.4	+0.91	[0.73; 1.09]	0.041	0.48
PAS (mm)	TB	10.6 ± 1.3	12.1 ± 1.4	+1.42	[1.27; 1.57]	<0.001	1.11
	RPE	10.5 ± 1.4	11.3 ± 1.3	+0.75	[0.47; 1.04]	0.022	0.59
	AMCOP^®^	10.4 ± 1.3	11.0 ± 1.3	+0.58	[0.36; 0.81]	0.037	0.46

## Data Availability

The data presented in this study are not publicly available due to privacy and ethical restrictions, as they consist of retrospective clinical radiographs and patient records. Anonymized data may be made available from the corresponding author upon reasonable request and pending approval by the institutional ethics committee.

## Abbreviations

Abbreviation	Definition
AHI	Apnea–Hypopnea Index
AMCOP^®^	Elastodontic orthodontic device
ANB	A point–Nasion–B point angle
ArGo–GoMe	Gonial angle
CBCT	Cone-Beam Computed Tomography
CE	Conformité Européenne
Co–Gn	Condylion–Gnathion mandibular length
FMA	Frankfort–Mandibular Plane Angle
GDPR	General Data Protection Regulation
IMPA	Incisor Mandibular Plane Angle
LL	Lateral cephalometric radiograph
MAS	Middle Airway Space
MDR	Medical Device Regulation
OSA	Obstructive Sleep Apnea
Overbite	Vertical incisal overlap
Overjet	Horizontal incisal overlap
PAS	Posterior Airway Space
RPE	Rapid Palatal Expander
SN–GoGn	Cranial base to mandibular plane angle
SNA	Sella–Nasion–A point angle
SNB	Sella–Nasion–B point angle
SPAS	Superior Posterior Airway Space
SPSS	Statistical Package for the Social Sciences
T0	Pretreatment time point
T1	Post-treatment time point
TB	Twin Block
